# The prevalence of lymphogranuloma venereum infection in men who have sex with men: results of a multicentre case finding study

**DOI:** 10.1136/sti.2008.035311

**Published:** 2009-02-15

**Authors:** H Ward, S Alexander, C Carder, G Dean, P French, D Ivens, C Ling, J Paul, W Tong, J White, C A Ison

**Affiliations:** 1 Imperial College London, London, UK; 2 Health Protection Agency Centre for Infections, UK; 3 Clinical Microbiology Department, University College London Hospitals NHS Foundation Trust, London, UK; 4 Brighton and Sussex University Hospitals, London, UK; 5 Mortimer Market Centre, Camden Primary Care Trust, London, UK; 6 Royal Free NHS Trust, London, UK; 7 Guys and St Thomas’s NHS Trust, London, UK

## Abstract

**Objective:**

To determine the prevalence of lymphogranuloma venereum (LGV) and non-LGV associated serovars of urethral and rectal *Chlamydia trachomatis* (CT) infection in men who have sex with men (MSM).

**Design:**

Multicentre cross-sectional survey.

**Setting:**

Four genitourinary medicine clinics in the United Kingdom from 2006–7.

**Subjects:**

4825 urethral and 6778 rectal samples from consecutive MSM attending for sexual health screening.

**Methods:**

Urethral swabs or urine and rectal swabs were tested for CT using standard nucleic acid amplification tests. Chlamydia-positive specimens were sent to the reference laboratory for serovar determination.

**Main outcome:**

Positivity for both LGV and non-LGV associated CT serovars; proportion of cases that were symptomatic.

**Results:**

The positivity (with 95% confidence intervals) in rectal samples was 6.06% (5.51% to 6.66%) for non-LGV CT and 0.90% (0.69% to 1.16%) for LGV; for urethral samples 3.21% (2.74% to 3.76%) for non-LGV CT and 0.04% (0.01% to 0.16%) for LGV. The majority of LGV was symptomatic (95% of rectal, one of two urethral cases); non-LGV chlamydia was mostly symptomatic in the urethra (68%) but not in the rectum (16%).

**Conclusions:**

Chlamydial infections are common in MSM attending for sexual health screening, and the majority are non-LGV associated serovars. We did not identify a large reservoir of asymptomatic LGV in the rectum or urethra. Testing for chlamydia from the rectum and urethra should be included for MSM requesting a sexual health screen, but serovar-typing is not indicated in the absence of symptoms. We have yet to identify the source of most cases of LGV in the UK.

Lymphogranuloma venereum (LGV) has re-emerged as a significant sexually transmitted infection (STI) among men who have sex with men (MSM) in the UK and Europe. Initial outbreaks and clusters of cases in The Netherlands, Germany, UK and France have been followed by reports in several other parts of Europe, North America and Australia.^1^ Before these outbreaks there were sporadic cases of LGV in the west, although it was probably under-diagnosed because of limited diagnostic methods and facilities. LGV was an established cause of proctitis in MSM but the prevalence and clinical spectrum of the infection were unknown.^2^ Previous outbreaks of LGV appear to have been relatively short lived, but there is increasing evidence that LGV has now become endemic among MSM in the UK. Between October 2004 and December 2008, the National Reference Centre identified LGV in 854 isolates (Health Protection Agency, unpublished data).

The clinical features of recent cases of LGV have been documented but there are many outstanding questions about the transmission and persistence of the infection in the population.^3^ ^4^ The overwhelming majority of cases have been rectal, with fewer cases diagnosed from urogenital sites. Reported risk factors for acquisition have included HIV infection, other STI, anal enema use, unprotected receptive anal intercourse, attendance at sex parties, reporting fisting and the use of sex toys.^5^ ^6^ These findings suggest that transmission could be predominantly rectal-to-rectal via intermediate carriage on hands or fomites such as toys or enema equipment. However, this does not explain transmission in those men who only report anal intercourse where the source is likely to be urogenital infection.

In the UK, if there was significant asymptomatic LGV this would not have been detected through routine channels, since LGV typing is only carried out on those with clinical syndromes suggestive of the infection. While urethral testing for chlamydia is now generally performed in clinics, rectal screening is not routinely done in all clinics.^7^ We therefore carried out a case finding exercise in which routine testing for chlamydia was standardised in four clinics in order to estimate the prevalence of LGV and non-LGV *Chlamydia trachomatis* (CT) in MSM in the UK, and to determine whether there is a significant amount of asymptomatic urogenital or rectal LGV infection that may be acting as a reservoir of undiagnosed and untreated infection.

## METHODS

Unselected MSM attending four genitourinary medicine (GUM) clinics in the UK for sexual health screening were tested for urethral and rectal CT regardless of symptoms. Data collection took place between 2006 and 2007 for limited time periods, usually three months, in each clinic. The clinics were chosen on the basis that they had already reported substantial numbers of cases of LGV and served large populations of MSM. They were already testing MSM for urethral chlamydia, but rectal testing was generally confined to those with symptoms. Before the undertaking of this initiative, CT-positive specimens were only forwarded to the reference laboratory for LGV typing if patients met the inclusion criteria—namely, having symptoms suggestive of LGV or being a contact of someone with LGV.

For the duration of the case finding exercise, all MSM attending each of the clinics had a rectal swab and a urine or urethral specimen taken for chlamydia testing, irrespective of symptoms but according to sexual risk assessment. Rectal and urethral/urine samples were tested in the local diagnostic laboratories using either the Probetec Strand Displacement Assay (SDA, Becton Dickinson, Sparks, MD, USA) or the Combas Amplicor (Roche Diagnostics Systems, Branchburg, NJ, USA) for the identification of CT. All samples that tested positive for CT were sent to the Sexually Transmitted Bacterial Reference Laboratory (STBRL) for LGV testing using an LGV-specific real-time polymerase chain reaction (PCR) assay.^8^ In one clinic, only positive rectal samples were forwarded for LGV typing.

Clinics recorded the numbers of urethral and rectal tests performed on MSM during the data collection period, and these were collated with results of chlamydia testing and LGV typing. For all cases of LGV, clinics were asked to confirm whether or not the patient had symptoms; for cases of non-LGV CT three clinics were also able to provide data on symptoms. Whether a patient was symptomatic was both patient reported (discharge, tenesmus, rectal bleeding, rectal pain, change in bowel habit, urethral symptoms) or based on clinical findings (proctitis, mucous discharge, contact bleeding seen on proctoscopy and sampling). Proctoscopy was performed in all patients.

Ethics committee approval and individual patient consent were not obtained as this was considered to be a standard case finding exercise in the context of an outbreak investigation; tests performed were in line with existing clinical guidelines; and routine testing for rectal chlamydia was standard care in many clinics already and recommended by BASHH guidelines of 2006.^7^ ^9^ Patients were informed of the tests that were being performed. Data on patients with LGV were collected retrospectively as part of case surveillance. Regardless of symptoms, all CT-positive specimens obtained at these centres were referred for LGV typing during this period and this was justified in the context of an epidemic where the rate of symptomatic LGV in London/Brighton was yet to be determined. Those with confirmed LGV-positive specimens had these results conveyed to them and were managed as appropriate at their respective clinics.

## RESULTS

Across the four clinics there were 4825 urethral/urine and 6778 rectal samples from MSM. In these samples the overall prevalence of CT was 3.25% in the urethra (157 positive tests) and 6.96% in the rectum (472 positive tests). Table 1 shows LGV and non-LGV CT positivity for urethral and rectal specimens overall and by clinic. The majority of CT at both anatomical sites was attributable to non-LGV strains (99.4% in the urethra and 87.1% in the rectum). Findings were consistent across the clinics.

**Table 1:** *Chlamydia trachomatis* (CT), lymphogranuloma venereum (LGV) and non-LGV chlamydia positivity in urethral and rectal specimens from MSM by clinic

Clinic*	Urethral chlamydia				Rectal chlamydia			
	% (95% CI)				% (95% CI)			
	No of tests	All CT	LGV	Non-LGV CT	No of tests	All CT	LGV	Non-LGV CT
A	3501	3.26 (2.67 to 3.85)	0.06 (0.01 to 0.14)	3.20 (2.62 to 3.78)	3501	7.21 (6.34 to 8.06)	1.09 (0.75 to 1.43)	6.11 (5.32 to 6.90)
B	933	3.43 (2.26 to 4.60)		3.43 (2.26 to 4.60)	1210	5.87 (4.55 to 7.19)	0.58 (0.15 to 1.01)	5.29 (4.03 to 6.55)
C	.		.	.	1672	7.18 (5.94 to 8.42)	0.60 (0.23 to 0.97)	6.58 (5.39 to 7.77)
D	391	2.81 (1.17 to 4.45)		2.81 (1.17 to 4.45)	395	7.34 (4.77 to 9.91)	1.52 (0.31 to 2.73)	5.82 (3.51 to 8.13)
All	4825	3.25 (2.78 to 3.80)	0.04 (0.01 to 0.16)	3.21 (2.74 to 3.76)	6778	6.96 (6.37 to 7.60)	0.90 (0.69 to 1.16)	6.06 (5.51 to 6.66)

*A, Brighton; B, Mortimer Market; C, Royal Free; D, St Thomas’s.

Clinical data were available for all cases of LGV and for all urethral non-LGV CT infections; clinical data were missing for 110/411 (26.8%) of rectal infections caused by non-LGV CT. The majority of LGV was symptomatic: 58 of 61 rectal cases (95%) and one of the two urethral cases. In centre A, two of the 38 cases of rectal LGV were also infected with gonorrhoea; one was asymptomatic and the other had discharge and proctitis. Details on co-infections were not available for other centres. Non-LGV chlamydia was mostly symptomatic in the urethra (105 of 155 cases, 67.7%) but not in the rectum (49/301, 16.3%).

## DISCUSSION

This large study in unselected MSM attending GUM clinics in London and Brighton shows an estimated prevalence of LGV of 0.90% in the rectum and 0.04% in the urethra, with little variation across the four centres included in this study. We did not identify a large reservoir of urethral LGV or of asymptomatic rectal LGV. We only identified two cases of urethral LGV and therefore it is not possible to make further generalisations about the presentation. The majority of cases of LGV in the rectum were in men with symptoms. Co-infection with gonorrhoea, for example, existed in a minority of cases in the one centre where this was documented. Non-LGV chlamydia was more prevalent and mostly asymptomatic in the rectum and symptomatic in the urethra.

This is the largest study to date to estimate the prevalence of LGV in MSM, and has the advantage of including men attending clinics serving different local populations. The study is limited by the lack of detailed data on all those who were screened, and therefore we are unable to provide a true prevalence estimate, since individuals may have been screened more than once, and we do not have details of symptoms for all those screened, or for some patients with chlamydia infection. The lack of these and related data such as HIV status reflect the nature of the study: it was established as part of an outbreak investigation with the aim of identifying undiagnosed cases that may help with control efforts.

A relatively small proportion of cases (6%) were asymptomatic, and it seems unlikely that such cases are a major factor in ongoing transmission. A linked study at another large clinic in London screened 3076 unselected MSM and found a similar overall prevalence of LGV with 36 cases (1.17%) including 35 rectal and one urethral.^10^ They report a higher proportion of asymptomatic rectal cases (17% compared with 5% in this study, this did not reach statistical significance p = 0.069). Taken together these results do not provide sufficient evidence to recommend routine screening of all MSM for rectal LGV. The current recommendation of diagnostic testing for CT with LGV-typing of CT-positive specimens in men with proctitis, or contacts of men with LGV, should remain in place. These findings contrast with reports from The Netherlands where the majority of LGV cases in some reports have been asymptomatic.^6^ ^11^ Our findings are from men attending GUM clinics rather than an unselected population sample, but this is unlikely to explain the discrepancy as they are similar to the settings for the research in The Netherlands.

**Key messages**

- There is a sustained outbreak of lymphogranuloma venereum (LGV) among men who have sex with men (MSM) in the UK.
- LGV was identified in 1% of rectal and <0.1% of urethral samples from MSM attending clinics.
- Non-LGV chlamydia was more common, found in 6% of rectal and 3% of urethral samples.
- Most LGV is symptomatic and no significant asymptomatic reservoir of infection has been identified.
- Testing for chlamydia from the rectum and urethra should be included for MSM requesting a sexual health screen, but serovar-typing is not indicated in the absence of symptoms.

We found very little urethral LGV which fits the general picture of the recent LGV outbreaks in Europe,^11^ but also poses the question of how transmission is being sustained. Early reports suggested transmission through sex toys or fisting, but more recent analyses have not supported this association, and more research into modes of transmission is required.^3^ ^4^ There is insufficient evidence to recommend routine screening of MSM for urethral LGV. Diagnostic tests for CT with LGV-typing of CT-positive specimens should be carried out on LGV contacts and men with signs of genital ulcer adenopathy syndrome or other suspected LGV disease. The question of how transmission occurs and what underpins the persistence of LGV will only be answered by more detailed clinical and epidemiological research.

We have confirmed previous reports of a significant prevalence of non-LGV rectal chlamydia in this population. In contrast to LGV, most of the rectal serotype D-K infection is asymptomatic. Guidelines from the United States, Canada and Australia recommend screening MSM for rectal chlamydia although they do not advise which test to use as none of the nucleic acid amplification tests (NAATs) are Food and Drug Administration (FDA) approved or CE marked for rectal specimens.^12^ In the UK there is no recommendation for routine rectal screening and the decision rests with local clinics and laboratories. Repeated studies of the performance of NAATs for CT on rectal samples have shown good validity, and in practice these tests are being increasingly being used within the UK.^7^ ^13^ The lack of approved tests applies equally to the issue of diagnostic testing, and UK guidelines indicate that NAATs are the test of choice in the diagnosis of chlamydial proctitis and LGV. This rather contradictory situation shows the urgency of developing a good evidence-based guideline on rectal chlamydia testing.

While the prevalence of rectal LGV is only 1%, it remains an important pathogen that can lead to severe acute symptoms and serious sequelae, and may facilitate the transmission of HIV and hepatitis C.^12^ The shift from sporadic outbreaks towards an endemic infection indicates that more needs to be done in terms of active case finding, with a low threshold of testing for chlamydia and LGV in MSM with symptoms, particularly in men with HIV, and an intensified health promotion campaign to alert MSM to the possibility of LGV infection.
